# Editorial: Central and peripheral mechanism interfering in metabolic syndrome

**DOI:** 10.3389/fendo.2023.1306261

**Published:** 2023-10-11

**Authors:** Mar Quiñones, Luisa María Seoane, Omar Al-Massadi

**Affiliations:** ^1^ Grupo Fisiopatología Endocrina, Área de Endocrinología, Instituto de Investigación Sanitaria de Santiago de Compostela, Complexo Hospitalario Universitario de Santiago (CHUS/SERGAS), Santiago de Compostela, Spain; ^2^ CIBER de Fisiopatología de la Obesidad y la Nutrición, Instituto de Salud Carlos III, Santiago de Compostela, Spain; ^3^ Translational Endocrinology Group, Endocrinology Section, Instituto de Investigación Sanitaria de Santiago de Compostela, Complexo Hospitalario Universitario de Santiago (IDIS/CHUS), Santiago de Compostela, Spain

**Keywords:** obesity, precocious puberty, SCFAs (short chain fatty acids), guanylin peptides, pancreas steatosis, macrophages, microglia

Substantial progress has been made in the last few decades in the understanding of the molecular pathways and physiological systems governing energy balance. While several metabolic diseases have approved treatments currently approved by regulatory bodies, the efficacy and/or safety of these is still a concern. It is therefore necessary to ascertain in depth the molecular mechanisms underlying these diseases and to identify new targets for therapeutic treatment. In line with this, the content of this Research Topic includes original papers and a review addressing central and/or peripheral mechanisms involved in disruptions of metabolic control in conditions such as obesity.

Obesity levels are constantly rising, having reached pandemic proportions worldwide; in fact, childhood obesity has doubled over the last decade. Childhood obesity, especially in girls, is frequently associated with earlier puberty onset, and may cause other metabolic complications later in life. The mechanisms underlying this association remain elusive. However, the implication of gut microbiota and its derived short chain fatty acids (SCFAs) as a link to both processes has been suggested.

In this context Wang et al. determine that in addition to an early gonadal maturation, obese rats presented a dysregulation in gut microbiota and the increased expression of some markers of the hypothalamic pituitary gonadal axis (HPGA). Moreover, they found that SCFAs added to the diet, in addition to reducing body weight in diet induced obesity (DIO) female rats can also ameliorate the symptoms of precocious puberty. At the molecular level, SCFA administration reverses the activation of the hypothalamic expression of genes associated with obesity-induced precocious puberty such as Kiss1, its receptor the protein G coupled receptor 54 and Gonadotropin releasing hormone (GnRH), probably by inhibiting the secretion of hypothalamic GnRH, which in turn reduces the incidence of precocious puberty.

Interestingly, the novelty and potential applications of these findings is discussed further in another article contained in the present Research Topic. In his commentary, Chen highlights the finding of SCFAs as playing a key role in obesity induced precocious puberty in female rats by regulating HPGA function. Moreover, he proposes that the hypothalamus could be a new target for SCFA action and describes a new gut-brain crosstalk pathway. Chen suggests the potential use of SCFAs as a novel alternative therapeutic intervention for precocious puberty in girls that could avoid the side effects of current medications. The beneficial effect of fibre administration to type 2 diabetes patients or the suggested use of prebiotic functional foods for treating obesity support this claim.

Obesity is moreover associated with ectopic fat accumulation in peripheral organs that could cause pancreatic β-cell dysfunction and insulin resistance which might ultimately led to type 2 diabetes developing. While changes in the levels of some endocrine factors such as the guanylin peptides in obesity or after bariatric surgery are well documented, the specific role of the pancreatic guanylin system in obesity was underestimated until now. In this regard, Otero et al. describe the involvement of the guanylin system in the improvement of pancreatic fat deposition in DIO rats after surgical or dietary manipulation. They found that surgical sleeve gastrectomy, in addition to promoting weight loss, reverses the inhibition of pancreatic guanylin and its receptor, the guanylate cyclase C induced by a high fat diet. Furthermore, they also describe a protective effect on pancreatic β-cell steatosis and insulin secretion of guanylin and uroguanylin in RIN-m5F rat insulinoma β-cells. This article suggests a new mechanism by which sleeve gastrectomy reduces fat accumulation in the pancreas and thus improves insulin resistance.

Obesity induces a proinflammatory state in peripheral organs such as the liver, pancreas and muscle that can act as an effector of different metabolic complications. However, obesity also induces a low-grade inflammatory state in the central nervous system (CNS) that can be ultimately associated with several metabolic traits such as dysregulation of the feeding circuitries. In this process, immune cells, such as neutrophils, eosinophils, and natural killer cells that act as a natural defence against potentially malignant infections play important roles. In respect to this, macrophages in the peripheral organs and microglia in the CNS are the main immune cells activated in obesity.

In the present Research Topic Mukherjee et al. extensively review the action and function of immune cells in metabolically active peripheral tissues. This is of paramount importance, due to the activity of these cells being able to stimulate the development of certain metabolic processes such insulin resistance, glucose control, energy expenditure or browning of white adipose tissue. Moreover, Mukherjee et al. present an in-depth and updated view of the role of the microglia in obesity and how they can modulate eating. Interestingly, they propose the small extracellular vesicles (sEV) as a novel potential mechanism of communication between the adipose tissue with the brain or with other peripheral organs. The authors conclude that although this is a new field of research, exploring the possibility of targeting these cell types or the use of sEV may be relevant to the development of new therapies to treat metabolic diseases.

To summarize, the articles assembled in this Research Topic will permit readers to draw on a more comprehensive range of conclusions with regard to the different processes associated with obesity.

## Author contributions

OA: Writing – original draft, Writing – review & editing. LS: Writing – original draft, Writing – review & editing. MQ: Writing – original draft, Writing – review & editing.

